# “Naturalization” of Routine Assisted Reproductive Technologies by In Vitro Culture of Embryos with Microvibration: Sex Ratio, Body Length, and Weight of 2,456 Live-Birth Deliveries after Transfer of 9,624 Embryos In Vitro Cultured in Static System and with Microvibration

**DOI:** 10.1155/2017/4964053

**Published:** 2017-12-20

**Authors:** Vladimir Isachenko, Karl Sterzik, Evgenia Isachenko, Robert Maettner, Plamen Todorov, Gohar Rahimi, Peter Mallmann, Erwin Strehler, Igor Pereligin, José Luis Alabart, Markus Merzenich

**Affiliations:** ^1^Research Group for Reproductive Medicine and IVF-Laboratory, Department of Obstetrics and Gynecology, Cologne University, Kerpener Str. 34, 50931 Cologne, Germany; ^2^Department of Reproductive Medicine, Christian-Lauritzen Institute, Frauenstr. 51, 89073 Ulm, Germany; ^3^Institute of Biology and Immunology of Reproduction, Tzarigradsko Shosse 73, 1113 Sofia, Bulgaria; ^4^IVF Center “Genesis Dnepr”, 119-120 Rybinska St., Dnipro, Ukraine; ^5^Servicio de Investigación Agroalimentaria (DGA), Avda. Montañana 930, 50059 Zaragoza, Spain; ^6^MedEvent Dr. Merzenich GmbH, Im Zollhafen 12, 50678 Cologne, Germany

## Abstract

Aim was to determine whether there is any difference in the sex ratio, body length, and body weight of 2,456 deliveries after transfer of 9,624 embryos derived using in vitro culture under static and mechanical microvibration conditions. Pronuclear embryos from 4435 patients were cultured in vitro under two different conditions: without (*n* = 4821) and with mechanical agitation (*n* = 4803). Sex ratio, body length, and weight of 2,456 live-birth deliveries after transfer of 9,624 embryos were noted. The proportion of males at birth was significantly associated with mode of in vitro culture of embryos only among women aged 40 years and older. The rate “body length” was significantly associated with mode of in vitro culture of embryos only among women aged 29 and younger. In the same time, among twins, this ratio positively associated with in vitro culture of embryos under microvibration only among women aged 30–34 years as well as ≥40 years and negatively among women aged 35–39 years. It was concluded that birth weight of infants was positively associated with mode of in vitro culture of embryos under microvibration among women of all age groups. This trial registration number is ISRCTN13773904, registered 6 April 2016.

## 1. Introduction

Epigenetic reprogramming is critical during gametogenesis and at preimplantation stage and involves DNA (deoxyribonucleic acid) methylation, imprinting, RNA (ribonucleic acid) silencing, covalent modifications of histones, and remodeling by other chromatin-associated complexes. Epigenetic regulation is involved in early embryo development, fetal growth, and birth weight. Disturbances in epigenetic reprogramming may lead to developmental problems and early mortality. There is concern about the health of children who are conceived with the use of assisted reproductive technologies (ART). It is reported that low birth weight and chromosomal anomalies can be associated with increased epigenetic disorders in the infants who are conceived using these procedures [1].

All fertilization steps in nature (receiving the ovulated oocytes, providing a suitable environment for fertilization) and embryo development with subsequent transport to the uterus take place inside the Fallopian tube. The tubal mucosa is arranged as longitudinal folds and consists of a single layer of cuboidal or columnar epithelium. The major cell types of this epithelium are secretory and ciliated cells [2], whose cilia have vibration or beating [3].

Vibration is a natural phenomenon that refers to mechanical oscillations about an equilibrium point. Since life began, the Earth has subjected all living things to a natural pulsation frequency. This natural phenomenon was predicted in 1952 [4] and named the global electromagnetic resonance phenomenon or Schumann resonances. Schumann resonances are quasi-standing electromagnetic waves that exist in the Earth's “electromagnetic” cavity (the space between the surface of the Earth and the ionosphere).

In medicine, the embryonic development rates and clinical results were compared between a static culture group (*n*  =  159 cycles) and a microvibration culture group (*n*  =  166 cycles) in poor responders. A microvibrator was set at a frequency of 42 Hz (Hertz, unit of frequency), 5 s/60 min duration human embryo development [5]. In poor responders, the embryo development rate was improved to a limited extent under the microvibration culture conditions, but the clinical results were significantly improved [6].

A retrospective analysis of a cohort of ICSI patients attending a German private fertility center during about 5 years was done. In vitro culture was performed either in a static environment with single oocyte/embryo culture (*n* = 291 patients) or under microvibration and group culture (*n* = 244 patients). In the static group, oocytes/embryos were cultured individually, while in the microvibration all the oocytes were cultured together and up to 4 embryos were cultured in the drop with a three-dimensional vibration of 56 Hz for 5 s/60 min. Authors have observed a significant increase in fertilization rate in oocytes cultured in groups and under microvibration conditions compared to oocytes cultured individually in a static culture (82% versus 78%), as well as in the implantation rate (42% versus 35%). In the same time, the authors have noted that the clinical pregnancy rate showed a tendency to be higher in the microvibration group but it did not reach significance (47% versus 43%) [6].

Earlier it was reported that in vitro culture of embryos under microvibration (with a mimic of conditions in nature whereby oviductal fluid is mechanically agitated by the epithelial cilia) significantly increases the quality of transferring embryos [7]. However, the data about peculiarities of newborn babies after transplantation of these embryos are limited.

The aim of our investigations was to determine whether there is any difference in the sex ratio, body length, and weight of 2,456 live-birth deliveries after transfer of 9,624 embryos derived using in vitro culture under static and mechanical microvibration conditions.

## 2. Materials and Methods

The authors confirm that all ongoing and related trials for this drug/intervention are registered (clinical trial registration ISRCTN13773904).

The manipulations with in vitro culture of embryos under microvibration were performed at a private medical center (Endokrinologikum Ulm, Praxisklinik Frauenstraße, Ulm, Germany, https://www.kinderwunsch-ulm.de/). Permission was granted by the Ethical Commissions of Medical Faculties of Ulm University, Germany (permission 321/10-UBB/bal. from 12.11.2011) and Cologne University, Germany (permission 13-147 from 20.11.2013) for the in vitro culture of embryos under mechanical microvibration. Couples were offered the choice of the in vitro culture of oocytes and embryos according to the standard routine or with mechanical agitation (microvibration) until transplantation. Written informed consent was obtained from all the participating couples.

Patients with infertility were stimulated for in vitro fertilization (IVF) cycle or intracytoplasmic spermatozoa injection (ICSI) cycle with triptorelin (Decapeptyl®, Ferring, Kiel, Germany) and recombinant FSH (Follicle stimulating hormone, Puregon®, MSD Sharp & Dohme GmbH, Haar, Germany or Menogon®, MerckSerono GmbH, Darmstadt, Germany, or Gonal-f®, MerckSerono GmbH, Darmstadt, Germany) according to the standard “short” protocol [8–10]. Ovulation was induced by the administration of 5000 IU (international unites) of hCG (human chorionic gonadotropin, Brevactid®, Ferring GmbH, Kiel, Germany) and oocytes were retrieved 34–36 hours later and inseminated with the partner's sperm through conventional IVF and ICSI techniques.

Patients were alternately assigned to the two embryo culture groups. Only two or three embryos per patient were cultured, as according to German law no more than three pronuclear oocytes/embryos from one patient (usually two) can be cultured in vitro and all cultured oocytes/embryos must be later transferred to the patient independently of the developmental rate of these embryos.

Oocytes for the culture of pronuclear embryos were obtained from 4435 subfertile/infertile couples. Informed patients were aged 26–46 years (median age 32.8). Pronuclear embryos (two or three per patient) were cultured in vitro under two different conditions: Group 1 (*n* = 4821), without mechanical agitation of the culture medium (standard routine conditions); and Group 2 (*n* = 4803), with mechanical agitation (44 Hz delivered over 5 s once every hour and acceleration (660 mV/g at 3.3 V: *X* = ±1.0 g, *Y* = ±0.7 g, *Z* = ±0.15 g)).

Mechanical agitation was achieved using the newly developed device Viboviduct 1500 (SimSoTec GmbH, Cologne, Germany, http://www.invitro360.com/). This device before using was calibrated by measurement of vibration with special device PCE-VT 2700 (PCE Instruments UK Ltd., Southampton, UK). Viboviduct 1500 generates microvibrations on the basis of special electric motor with low electromagnetic noise. The vibration generator is isolated from the corpus of machine for elimination of vibration of whole corpus. This vibration generator transfers vibrations to the carrier for Petri dishes with using only one element connected carrier and vibration generator. In that way, the generated vibrations are forwarding directly to the plate with Petri dishes. An electronic display for control of the electric vibration parameters is located on the corpus of machine. The use of the apparatus presupposes the election of certain operating program with speed of rotation, which determines the vibration frequency. The vibration generator is equipped by the internal EMC (Electromagnetic Compatibility) filter. This filter reduces electromagnetic interference by removing some high frequency noises signals in diapason from 100 KHz to 100 MHz. Petri dishes with embryos are fixed on the plate. The device is designed and developed for use in CO_2_-incubator.

Parameters of frequency identification, delay, and duration can be changed by operator. In order to have the same characteristics for all producing devices, each device is calibrated after manufacturing, before realization. Quantity of revolutions (rotations) per minute (abbreviated rpm, RPM, rev/min, r/min) is the number of rotations around a fixed axis in one minute. The disc rotation at 60 rpm with 2*π* radius per second means the frequency 1 Hz: 1 rpm = 1/60 Hz. In that way, with 2640 rpm, a frequency can be calculated as 44 Hz (2640 rpm per 60 seconds).

The embryos were cultured in 50 *μ*l of culture medium (Sage, Los Angeles, CA, USA) [11] under mineral oil (Sigma, St. Louis, MO, USA) to their transfer. For culture of embryos from 1 to 3 days Quinn's Advantage Cleavage media were used composed of 0.1 mM glucose, 3.9 mM lactate, 0.52 mM pyruvate, 2.2 mM calcium, 4.7 mM potassium, 118 mM chloride, 132 mM sodium, 1.8 mM magnesium, 1 *μ*g/L iron, 18 *μ*g/L aluminum, 0.2 *μ*g/L chromium, 0.2 *μ*g/L cobalt, and 3.6 *μ*g/L manganese. This medium was not supplemented by essential and supplemented by nonessential amino acids [11].

For culture from 3 to 5 days, Quinn's Advantage Blastocyst media were used, supplemented by essential and nonessential amino acids as well as by 2.8 mM glucose, 0.16 mM citrate, 3.9 mM lactate, 0.07 mM pyruvate, 2.2 mM calcium, 0.3 mM phosphorus, 4.9 mM potassium, 113 mM chloride, 132 mM sodium, 1.8 mM magnesium, 2 *μ*g/L iron, 21 *μ*g/L aluminum, 0.2 *μ*g/L chromium, 0.2 *μ*g/L cobalt, and 3.6 *μ*g/L manganese [11].

Pregnancy was defined as an increase in serum hCG concentration (20 IU/L) determined on 11 and 13–15 days after embryo transfer. Clinical pregnancy was recorded when the fetal sac was visualized on an ultrasound on gestational weeks seven to eight.

The following three parameters of newborn infants were evaluated: sex, length of body, and weight of body. For evaluation of body length, distribution on three groups was performed: <48 cm, 48–54 cm, and >54 cm. All data regarding weight of body were also distributed on three groups: <2800 grams, 2800 to 3800 grams, and >3800 grams.

### 2.1. Statistical Analysis

The percentages of babies taken at home and of masculine sex were analyzed by ANOVA for categorical variables using the CATMOD (categorical modelling procedure) of SAS Institute Inc. [12]. The terms included in the model were culture method (vibration versus static) and interaction inside of respective groups. Between-groups comparisons were performed by pairwise contrasts. Probabilities were corrected for multiple comparisons by the Bonferroni-Holm method using the MULTTEST procedure of SAS. The level of statistical significance was set at *P* < 0.05.

## 3. Results

### 3.1. Sex of Infants

Ratio “sex of newborn infants” in respective groups generally can be characterized as invariable. The differences in this ratio of newborn infants among women of respective groups aged ≤ 29 years, 30–34 years, 35–39 years (2222 children), and >40 years (234 babies) were not established (*P* > 0.1) (Table 1).

The maximum difference between culture methods was observed in the oldest (>40 years) group (58.1 versus 46.6% in static and vibration groups, resp.). However, significance was not achieved (*P* > 0.1) due to the lower number of babies involved in this age group. In addition, this difference was found to be clearly nonsignificant when correction for multiple comparisons was performed (*P* > 0.1).

### 3.2. Body Length

**Figure 1:**
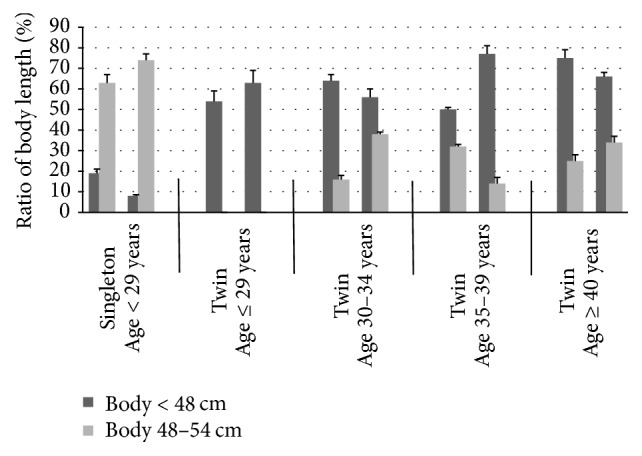
Length of body of newborn babies after transplantation of embryos in different age groups: in vitro culture in static system and with microvibration. Only presented here are significant differences (*P* < 0.05); rest of respective ratios of body length in “Static” and “Vibration” groups are similar (*P* > 0.1).

Ratio “length of body of newborn babies” after transplantation of embryos in different age groups after in vitro culture in static system and with microvibration is more variable than the rate “sex of newborn infants” (Figure 1).

The absence of differences in this ratio was observed among singletons quasi in all age groups excluding groups of women aged ≤ 29 years. Babies with body length < 48 cm were born 19% in “Static” group and 8% in “Vibration” group (*P* < 0.05). Babies with body length 48–54 cm was born 63% in “Static” group in contrast with 74% in “Vibration” group (*P* < 0.05, Figure 1).

More differences were observed in this ratio for “twin” women. Babies with body length < 48 cm were born in “Static” group comparatively to “Vibration” group, respectively: 54% and 63% by women aged ≤ 29 years, 64% and 56% by women aged 30–34 years, 50% and 77% by women aged 35–39 years, and 75% and 66% by women aged ≥ 40 years (*P* < 0.05, Figure 1).

Among twins, the absence of differences in this ratio was observed for all age groups of women. Percent of babies with body length < 48 cm in “Static” group comparatively to “Vibration” group was, respectively, 16% and 38% by women aged 30–34 years, 32% and 14% by women aged 35–39 years, and 25% and 34% by women aged ≥ 40 years. Among twins, no baby born with body length > 54 cm was observed (*P* < 0.05, Figure 1).

Among triplets, babies with body length < 48 cm were born in both “Static” and “Vibration” groups by women aged 30–34 years (*P* > 0.1) and only in “Static” group by women aged 35–39 years (*P* < 0.05, Figure 1).

In such way, among singletons, the rate “body length at birth” was significantly associated with mode of in vitro culture of embryos only among women aged 29 and younger: percent of babies with body length 48–54 cm in “Static” group comparatively to “Vibration” group was 63% and 74%, respectively. In the same time, among twins, this ratio positively associated with in vitro culture of embryos under microvibration only among women aged 30–34 years as well as ≥40 years and negatively among women aged 35–39 years (*P* < 0.05, Figure 1).

### 3.3. Body Weight

**Figure 2:**
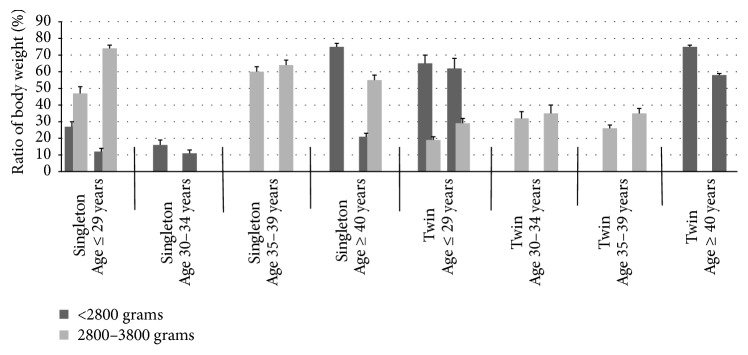
Body weight of newborn babies after transplantation of embryos in different age groups: in vitro culture in static system and with microvibration. Only presented here are significant differences (*P* < 0.05); rest of respective ratios of body weight in “Static” and “Vibration” groups are similar (*P* > 0.1).

Among singletons, it was established that the ratio “body weight of newborn babies” in “Static” group of women aged 35–39 years has no differences from this ratio in “Vibration” group (*P* > 0.1, Figure 2). Percent of babies with body weight 2800 to 3800 grams in groups of women aged 30–34 years was similar in both “Vibration” and “Static” groups (*P* > 0.1, Figure 2).

Among singletons, percent of babies with body weight < 2800 grams in “Static” group comparatively to “Vibration” group was, respectively, 27% and 12% by women aged ≤ 29 years, 16% and 11% by women aged 30–34 years, and 75% and 21% by women aged ≥ 40 years (*P* < 0.05, Figure 2).

This ratio was more invariable among twins. Percent of babies with body weight < 2800 grams in “Static” group comparatively to “Vibration” group was, respectively, 65% and 62% by women aged ≤ 29 years and 75% and 58% by women aged ≥ 40 years (*P* > 0.1, Figure 2).

Among twins, percent of babies with body weight 2800 to 3800 grams was similar in both “Vibration” and “Static” groups for women aged 30–34 years and ≥40 years (*P* > 0.1, Figure 2).

Percent of babies with body weight 2800 to 3800 grams in “Static” group comparatively to “Vibration” group was, respectively, 19% and 29% by women aged ≤ 29 years and 26% and 35% by women aged 35–39 years and 75% and 21% by women aged ≥ 40 years (*P* < 0.05, Figure 2).

No baby born with body ≥ 3800 grams was observed among twins (Figure 2).

Among triplets, babies with body weight < 2800 grams were born only in both “Static” and “Vibration” groups by women aged 30–34 years (Figure 2).

In such way, birth weight was positively associated with mode of in vitro culture of embryos under microvibration among women of all age groups.

## 4. Discussion

The authors would like to note that new IVF-technology presented here is being used without much knowledge on the long-term consequences on the progeny in contrast with “standard” IVF-technology which presupposes the in vitro culture of oocytes and embryos in static conditions.

In described treatments, two groups of 4435 patients were subjected for in vitro fertilization (IVF, 1153 patients, 26%) and intracytoplasmic spermatozoa injection (ICSI, 3282 patients, 74%). The programming statistical analysis of our results has demonstrated that correlations between type of ART treatment (IVF of ICSI) and effect of microvibration on the quality of embryos and some peculiarities of newborn babies were not found. It was the ground to present the whole information together, independently from type of ART treatment (IVF or ICSI).

There is concern about the health of children who are conceived with the use of assisted reproductive technologies (ART) [1]. The purpose of study of Klemetti et al. [13] was to examine the health of 4559 children who were born as a result of in vitro fertilization. Perinatal outcomes of in vitro fertilization children were worse and hospital episodes were more common than among control children. Risks for cerebral palsy and psychological and developmental disorders were increased [13].

Maalouf et al. [14] have investigated the effect of ART treatments on the sex ratio of babies born: a total of 106,066 babies of known gender born from 76,994 mothers after 85,511 IVF-treatments between 2000 and 2010 in the United Kingdom. It was established that ART lead to different sex ratios, highest after IVF and lowest under ICSI embryo transfer [14].

Routine ART today involve the use of static culture systems. However, in natural conditions (in vivo), the egg, spermatozoon, and embryo are subjected to ever changing dynamic processes.

Mechanism of in vivo fertilization and following development of embryos is the complex involving the concerted actions of motile spermatozoa as well as ciliary beating just from the moment of ovulation and fertilization and during embryo transport via the oviduct to the uterus [15].

Initially, the information reporting the benefits of pulsative mechanical microvibration for the cytoplasmic maturation of in vitro matured pig oocytes was published [16]. These authors subjected cumulus-oocyte-complexes cultured in microdrops to pulsatile mechanical vibration at a frequency of 20 Hz with acceleration (660 mV/g at 3.3 V: *X* = ±1.0 g, *Y* = ±0.7 g, *Z* = ±0.15 g; instruction of manufacturer). During in vitro maturation, vibration did not affect the proportion of oocytes reaching the Metaphase-II stage. However, blastocyst formation rates after the activation of oocytes exposed to vibration were significantly higher than those obtained for oocytes matured without mechanical vibration (27% versus 12% and 26 versus 15%, resp., for the 5 s and 10 s pulses).

Mechanical vibration applied to somatic cells cultured in vitro activates their proliferation and secretory properties. Observations include (i) the enhanced proliferation of bovine articular chondrocytes [17]; (ii) the time-dependent augmentation of DNA synthesis and also the promotion of proteoglycan synthesis during long-term culture in response to periodic vibration at a frequency of 300 Hz [18]; and (iii) increased interleukin-8 release in human bronchial epithelial cells [19]. In addition, vocal fold-like vibrational stimuli have been found to influence the expression of several key matrix and matrix-related genes, enhance the secretion of the profibrotic cytokine transforming growth factor, increase the accumulation of the extracellular matrix proteins, fibronectin, and collagen type 1, stimulate vocal mucosa-like matrix expression by hydrogel-encapsulated fibroblasts, and enhance construct stiffness compared with nonstimulated controls [20, 21].

It was established that mechanical stimuli differentially influence factors involved in the induction of angiogenesis. Intercellular communication enables cells to coordinate their physiological activity and establish cell cooperation. This confers on cell systems the ability to respond uniformly to a localized stimulus as an important response in cell behavior and differentiation [22–24].

It was clearly shown that mechanical stimulation of ciliated epithelial cells in culture induces a wave of increasing Ca^2+^ that spreads from the stimulated cell to neighboring cells. In the absence of extracellular Ca^2+^, these mechanically stimulated cells showed no change or a decrease in [Ca^2+^], whereas [Ca^2+^] increased in neighboring cells. Additionally, iontophoretic injection of inositol 1,4,5-trisphosphate (IP3) in treated epithelial cells in culture evoked a communicated Ca^2+^ response that was similar to that produced by mechanical stimulation. These data allowed these authors to postulate that IP3 acts as a cellular messenger that mediates communication through gap junctions between ciliated epithelial cells [25].

Taking into account the fact that for “naturalization” of routine ART we have used the culture of embryos under microvibration, the aim of our investigations was to determine whether there is any difference in the sex ratio, body length, and weight of live-birth deliveries after transfer of embryos derived using in vitro culture under static and mechanical microvibration conditions.

## 5. Conclusion

The proportion of males at birth was significantly associated with mode of in vitro culture of embryos only among women aged 40 years and older: in “Static” and “Vibration” groups 50% and 40%, respectively.

Among singletons the rate “body length at birth” was significantly associated with mode of in vitro culture of embryos only among women aged 29 and younger: percent of babies with body length 48–54 cm in “Static” group comparatively to “Vibration” group was 63% and 74%, respectively. In the same time, among twins this ratio positively associated with in vitro culture of embryos under microvibration only among women aged 30–34 years as well as ≥40 years and negatively among women aged 35–39 years.

Birth weight of infants was positively associated with mode of in vitro culture of embryos under microvibration among women of all age groups.

## Figures and Tables

**Table 1:** Sex of new born babies after transplantation of embryos in different age groups: in vitro culture in static system and with microvibration.

Age of patients (newborn babies)	In vitro culture in static system				In vitro culture with microvibration			
	≤29 years (149 babies)	30–34 years (408 babies)	35–39 years (403 babies)	>40 years (100 babies)	≤29 years (163 babies)	30–34 years (543 babies)	35–39 years (556 babies)	≥40 years (134 babies)
“Baby-take home” rate	30%^a^	28%^b^	23%^d^	9%^f^	31%^a^	37%^c^	29%^e^	15%^g^
Masculine	39%	45%	46%	50%	40%	47%	46%	40%
Feminine	45%	42%	43%	36%	48%	43%	44%	46%

Different superscripts indicate significant difference (*P* < 0.05) between the respective rate in “Static” and “Vibration” groups; rates without superscripts indicate no significant difference (*P* < 0.05) between the respective rate in “Static” and “Vibration” groups (*P* > 0.1); the sum of percentages in each age group is less than 100, because the rest of data are unknown.
